# Applications of image-guided locoregional transarterial chemotherapy in patients with inoperable colorectal cancer: a review

**DOI:** 10.3389/fonc.2024.1464242

**Published:** 2024-08-23

**Authors:** Wenjun Meng, Lu Pan, Li Huang, Qing Li, Yi Sun

**Affiliations:** ^1^ Department of Biotherapy, Cancer Center, West China Hospital, Sichuan University, Chengdu, China; ^2^ West China School of Medicine, Sichuan University, Chengdu, China; ^3^ West China School of Nursing, Sichuan University, Chengdu, China; ^4^ Department of Oncology and Hematology, Air Force Hospital of Western Theater Command, Chengdu, China

**Keywords:** transarterial chemotherapy, chemoembolization, colorectal cancer, neoadjuvant therapy, comprehensive treatment

## Abstract

With the development of comprehensive treatment, locoregional transarterial chemotherapy has become an alternative conversion therapy, palliative therapy, and neoadjuvant therapy for many solid malignant tumors. Locoregional transarterial chemotherapy, which is most frequently used for treating liver cancer, has the characteristics of high regional efficacy and few systemic adverse reactions. In recent years, the number of relevant reports of locoregional chemotherapy for treating initially inoperable colorectal cancer (CRC), including non-metastatic and metastatic CRC, has gradually increased. However, the specific treatment options for such locoregional therapy are not the same, and its indications, medication regimens and combined treatments have not reached any consensus. In this review, the application status of locoregional transarterial chemotherapy in primary and metastatic CRC patients has been reviewed and summarized to provide a reference for future clinical work and scientific research.

## 1 Introduction

Colorectal cancer (CRC) is a common disease of the human digestive system with difficulties in the diagnosis and treatment (1). According to the global cancer statistics released, the current incidence and mortality of CRC rank third and second among all malignant tumors, respectively (2). With the global improvement of economies and living conditions, the number of newly diagnosed CRC patients is showing a rising trend, especially in developing countries. As the largest developing country, China is facing a more severe cancer burden due to backward cancer screening (3). The incidence of CRC in China has increased rapidly in the past twenty years and has reached 17.81/100,000 (4). If early detection and treatment are not available, such CRC patients may already be in the moderate or advanced stage when clinical symptoms appear. Patients with local invasion or distant organ metastasis (clinical stage III-IV) always have an extremely shorter survival time owing to the rapid progression of the primary disease and distant metastasis (DM). The five-year survival rate of CRC patients with DM is approximately about 10%, while in CRCs with localized lesions, the figure exceeds 90% (5–7).

At present, the radical cure for most CRC patients is surgical resection of the primary lesion with adjacent lymph nodes. Patients treated with surgery have obvious advantages in terms of quality of life and prognosis, compared to those treated with only drugs or palliative care. Once the tumor is evaluated as stage I or II, direct tumor excision is mostly regarded as the first choice without any neoadjuvant treatment (8). Due to the dominant position of surgical principles in CRC, the purpose of most preoperative treatments is to create operable conditions for surgeons. However, only a small number of CRC patients can undergo surgery in the initial stage because of local tumor infiltration, lymph node metastases and DM, etc. In the process of CRC treatment, the use of multidisciplinary team (MDT) is widespread, and long-term benefits have been confirmed after MDT discussions (9, 10). The satisfactory effect of MDT should be attributed to the rational application of various systemic and regional strategies in those patients, such as chemotherapy, radiotherapy, hormone therapy, biotherapy, ethnomedicine therapy, and interventional therapy, etc. (11) As a result, the concept of CRC treatment has been transformed into a pattern of combining multiple modes under the guidance of MDT with surgical treatment at the core.

Since CRC is a local lesion at the macro level, studies on locoregional therapies for CRC have never stopped. With the progress of interventional medicine, minimally invasive interventional chemotherapy has been included into the comprehensive treatment of various advanced solid tumors, and has achieved good results (12–16). Among them, liver cancer especially hepatocellular carcinoma (HCC) is the most widely applied kind of malignant tumors. In the 1970s, transcatheter arterial chemoembolization (TACE) was first proposed for liver cancer treatment using Seldinger technology and has been actively spread clinically (17). With more than 40 years of development of new drugs and materials, hepatic arterial infusion chemotherapy (HAIC) and TACE combined with other therapies have been used throughout the treatment of both primary and secondary liver cancer (18). Currently, TACE has become the first-line method for advanced HCC; indeed, it is considered the best choice for inoperable primary HCC (19). For secondary liver cancer, which is liver metastatic tumor, HAIC and TACE are also optional methods when surgical operation is not applicable for the liver lesion. Liver is the most common metastatic organ of CRC, and colorectal liver metastasis (CRLM) is also the main cause of cancer-related death (20). Statistics indicate that approximately 15% to 25% of CRC patients have liver metastasis at the time of initial diagnosis, and about 10% to 25% of CRC patients will ultimately develop CRLM after the primary lesion is resected (21–23). Although DM is often regarded as a contraindication to surgery, the best treatment for CRLM is resection of both the primary lesions and liver lesions if possible, as well as liver transplantation (24, 25). For CRLM patients with liver lesions already resected, the 5-year survival rate can be greater than 30%, while for patients without liver lesions resected, the 5-year survival rate can be less than 20%; however, there are only about 20% of patients with CRLM whose liver metastases can be surgically treated after the initial evaluation (26, 27). If conversion therapies for CRLM patients with unresectable liver metastases are successfully applied to reduce metastases to potentially resectable status, followed by surgery, the 5-year survival rate can be improved to the same level as that of CRLM with initially liver lesion resectable (28, 29). Therefore, multiple conversion therapies must be used to convert liver lesions to a resectable state, so as to improve the prognosis of CRLM. Normal hepatic tissue is mainly supplied by the portal vein, while the hepatic artery accounts for only approximately one-quarter of hepatic tissue; however, after the formation of liver metastases, the blood supply from the hepatic artery increases to 90% or more, which is also the anatomical basis for locoregional transarterial chemotherapy (HAIC and TACE) of hepatic lesions (30–32). The 5-year survival of CRLM has gradually increased in recent decades due to locoregional chemotherapeutic treatments.

**Figure 1:**
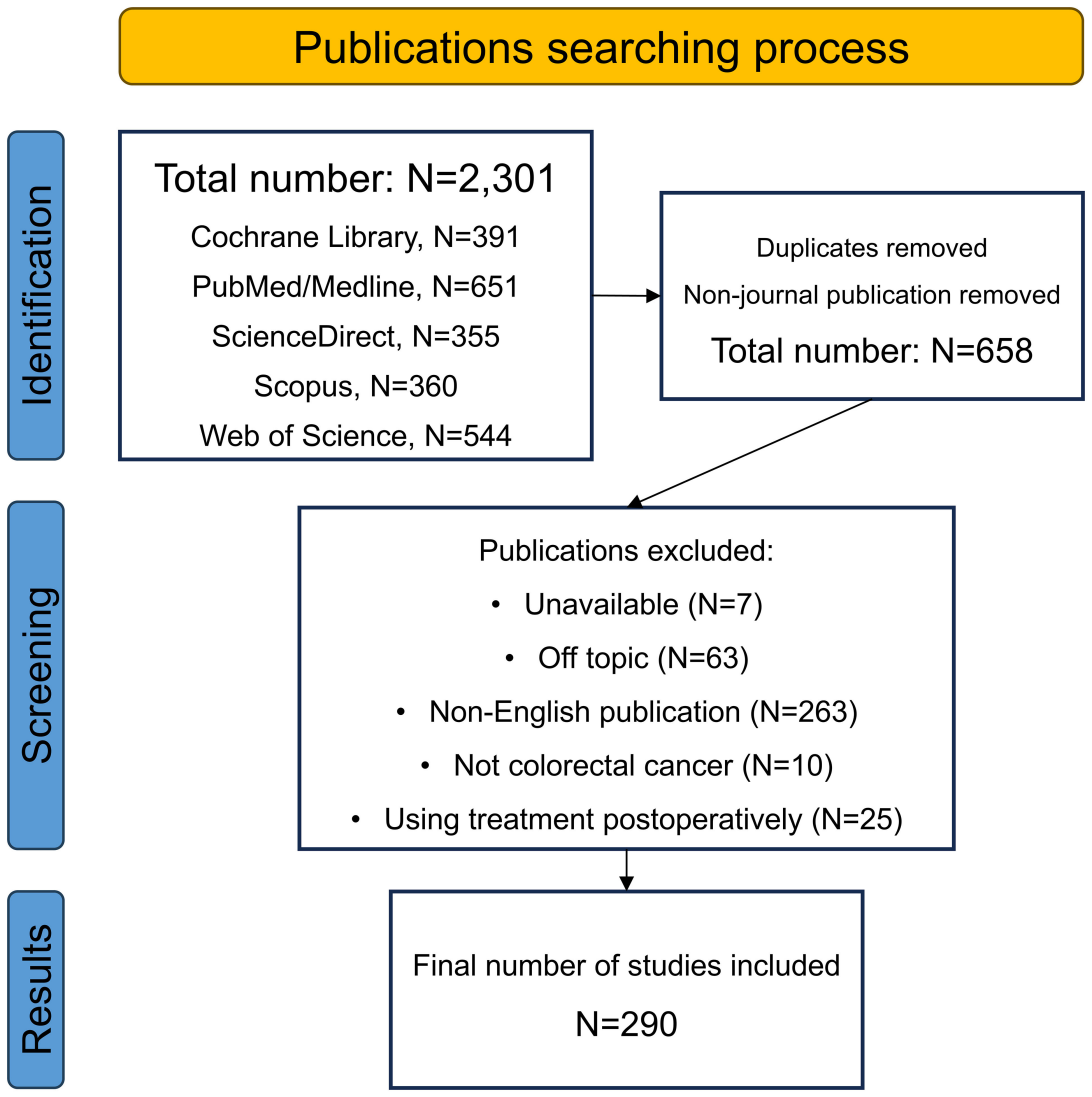
The flowchart of the searching process.

Apart from its universal use in liver cancer, interventional chemotherapy has also been actively applied in many other gastrointestinal cancers. For most advanced patients with gastric cancer (GC) or pancreatic cancer (PC), radical surgery is not applicable generally due to vast vascular involvement. Thus, conversion therapy including interventional chemotherapy must initially be used to improve the operation rate and R0 resection rate (33). Previous studies have demonstrated that interventional chemotherapy is an effective approach as both conversion and palliative treatment for advanced GC patients, and has a lower toxicity and complications than systemic chemotherapy (34, 35). Besides, when HAIC is used jointly with systemic chemotherapy in advanced PC, the response rate and survival are also improved (36, 37). However, reports of locoregional chemotherapies for primary CRC lesion are still rare. The good results in GC, PC and liver tumors have stimulated related studies in the CRC field. Recently, several researchers have performed large-scale investigations on the use of locoregional chemotherapy in CRC patients. To summarize recent updates of the locoregional transarterial chemotherapy and chemoembolization used for treating CRC in both localized and liver-metastatic lesions, we conducted this review by searching for data in electronic databases of Cochrane Library, PubMed/Medline, ScienceDirect, Scopus, and Web of Science. **Figure 1** displays the flowchart of the searching strategy and process.

## 2 Hepatic arterial infusion chemotherapy for unresectable colorectal liver metastases

Serving as a palliative, conversion and adjuvant therapy, and using Seldinger technology and digital subtraction angiography (DSA), HAIC has become a common method for locoregional chemotherapy in patients with CRLM (38–41). During this process, chemotherapeutic drugs are precisely delivered to the supply arteries of liver metastatic lesions via catheters and microcatheters, after which the tumor can be specifically attacked. Through local puncture via the femoral or radial artery, the tumor-feeding arteries are superselected. Then, chemotherapeutic drugs at high concentrations directly reach the feeding artery without any metabolism and fully contact the tumor tissue, and the targeted cells are killed immediately. This method can increase the concentration of locoregional drugs by dozens of times and has lower systemic toxicity (42–44). Since tumors are very sensitive to the concentration of chemotherapeutic drugs, the multiplication of drug concentration around tumors will correspondingly improve the effect of chemotherapy. A pharmacokinetics study also indicated that when the concentration of local administration is doubled, the killing intensity of the targeted tumor cells can increase by more than ten times (45).

However, the use of HAIC alone in patients with CRLM remains controversial. For example, a case-control study showed that the median overall survival (OS) of CRLM patients treated with fluorouracil-based HAIC combined with systemic chemotherapy was extended to 32.8 months, while the median OS of patients treated with systemic chemotherapy alone was only 15.3 months, indicating a significant statistical difference (46). Additionally, a recent meta-analysis indicated that HAIC as a palliative method achieved a greater tumor remission rate for liver metastatic lesions in CRLM than did systemic chemotherapy, but there was no significant improvement in either progression-free survival (PFS) or OS (47). Therefore, most clinical institutions around the world have abandoned the use of HAIC alone in CRLM patients, and the combination of HAIC and systemic chemotherapy as well as targeted therapy has gradually become one of the directions of current applications in order to maximize its efficacy. A completed phase 2 study using HAIC with floxuridine plus systemic oxaliplatin and irinotecan reached an overall response rate (ORR) of 92% in 49 CRLM patients, with a median OS of 39.8 months (48). Another recent phase 2 study reported that HAIC using oxaliplatin plus systemic 5-fluorouracil (5-Fu) with cetuximab achieved an ORR of 88% in patients with 35 frontline CRLM patients; and the median PFS and OS were 17.9 and 46.3 months, respectively (49). However, other large-scale prospective trials on HAIC plus systemic therapy are still in the initial stage, so there is a lack of studies on the standard use of HAIC plus systemic therapy for CRLM (50).

As shown above, the drug selection is another controversy. Traditional chemotherapeutic agents, such as fluorouracils, oxaliplatin, and raltitrexed etc., are the most commonly used drugs for arterial infusion with their monotherapy or combination (51–53). In the United States, fluorouracils [5-Fu, irinotecan, and fluorodeoxyuridine (FUDR), etc.] are considered the most widely used drugs for HAIC (54). However, 5-Fu might be replaced because it has a longer arterial infusion time with a similar efficacy compared to other drugs (52, 55). One of the first-line systemic chemotherapy drugs for treating CRLM is oxaliplatin, which is the most commonly used drug in European countries, and it requires only approximately 2 hours for infusion; furthermore, oxaliplatin has shown efficacy as a HAIC drug in patients who are resistant to oxaliplatin-based systemic chemotherapy (56, 57). Recently, the anti-angiogenic agent bevacizumab was also reported to be useful for hepatic arterial infusion, and may be an interesting option for the second-line treatment of CRLM patients (51, 58, 59).

## 3 Transcatheter arterial chemoembolization for unresectable colorectal liver metastases

**Figure 2:**
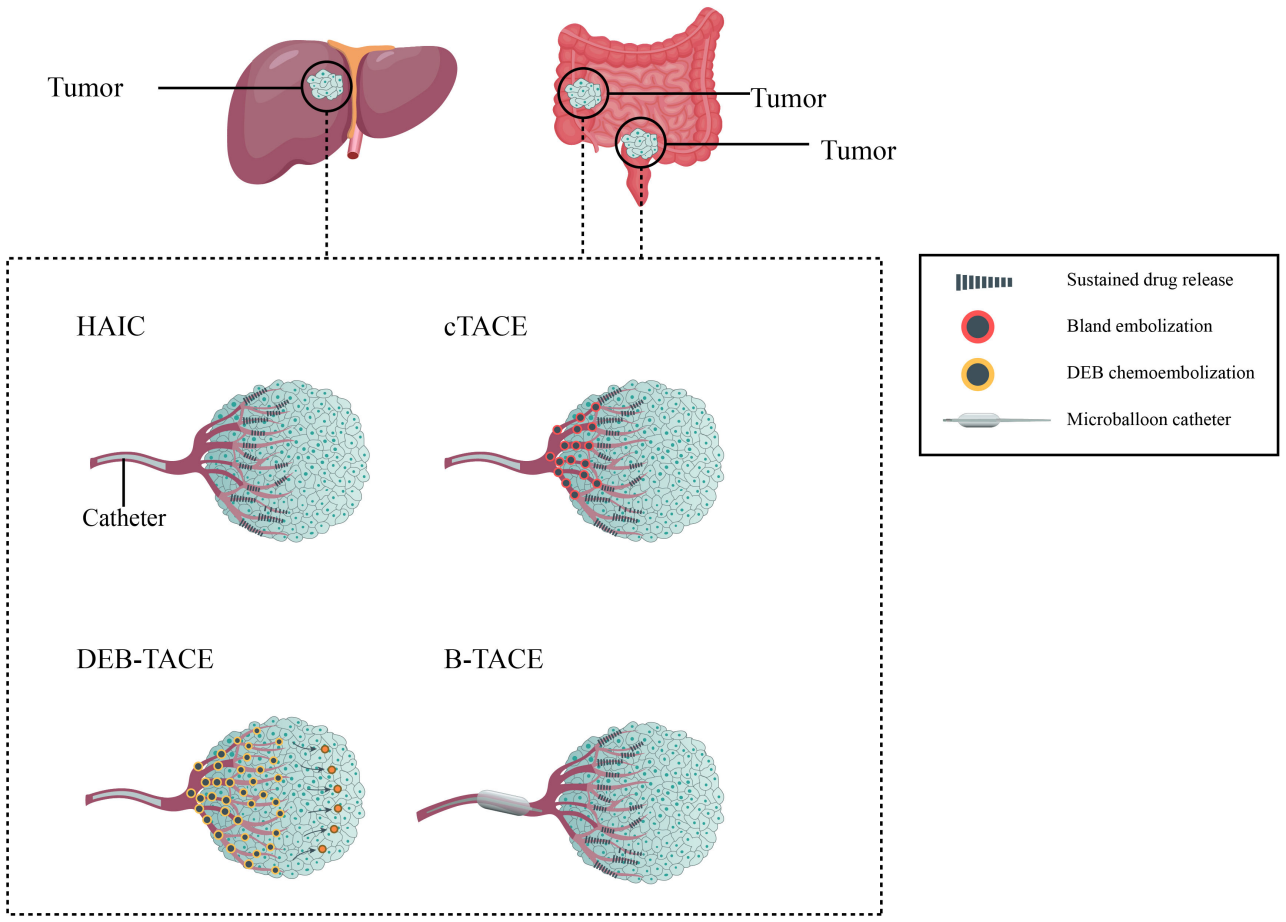
Different type of locoregional transarterial chemotherapy in primary and metastatic colorectal cancer patients.

The addition of embolization after infusion chemotherapy is a characteristic of TACE compared to HAIC. After the completion of drug infusion, the tumor feeding arteries are subsequently embolized with commonly used embolic agents, such as gelatin sponges, lipiodol, or drug-eluting beads (DEBs), etc. in the end (18, 60–62). According to different embolizing materials used, the TACE technique mainly includes conventional TACE (cTACE) and DEB-TACE (63). Additionally, new materials have developed new strategies such as balloon-occluded TACE (B-TACE), which expand the range of TACE technique and show promising results in primary HCC (64). **Figure 2** shows different type of locoregional transarterial chemotherapy in primary and liver metastatic CRC patients. The embolizing procedure of TACE can increase the drug contact time and drug concentration in the tumor tissue, resulting in more powerful cytotoxicity and ischemic necrosis of the tumor tissue; meanwhile, the drug concentration in surrounding normal tissue can be correspondingly decreased (18, 25, 60, 65–67).

However, no evidence has shown that TACE has better benefits in CRLM compared with HAIC, although TACE is a standard therapy. Indeed, there are reports on the combination of TACE and HAIC in treating liver tumors of CRLM, which may be another option when first-line therapy fails (68–70). Due to the controversy regarding the choice of cTACE or HAIC, DEB-TACE has become a promising star (71). DEB allows for the sustained and targeted release of chemotherapeutic agents directly to the tumor site, potentially enhancing the drug concentration within the tumor. DEB also prolongs the drug release time and enhances the effectiveness of the embolization procedure. Several small-scale studies have shown that the addition of DEB-TACE is reliable for treating liver lesions of CRLM, and the incidence of adverse events was in a low level even if more than three cycles of DEB-TACE were administered (72, 73). Although DEB-TACE has been widely carried out, there is no unified standard for the selection of loading drugs, embolic agents or catheter specifications, and its treatment endpoints are still controversial (74). The most commonly used loading drugs contain irinotecan and doxorubicin, and raltitrexed-loaded DEB has also been reported (75). However, no large studies have been finished yet, resulting in the starving for a direct comparison between DEB-TACE and cTACE in CRLM patients. Recently, Li et al. retrospectively analyzed a small sample of CRLM patients after multi-line therapies (76). DEB-TACE and cTACE were applied in a total of 22 patients, and the results indicated that 11 patients who received 300–500μm DEB-TACE had significantly longer PFS and OS than the other 11 patients who underwent cTACE. While DEB-TACE has shown benefits, there is limited evidence, especially large-scale studies, demonstrating a clear survival advantage of DEB-TACE compared to cTACE. Moreover, DEB is more expensive than conventional embolic agents, contributing to higher overall procedural costs.

Compared with the commonly accepted DEB specifications of 100–300μm or 300–500μm in diameter, recent studies have suggested that smaller specifications of 30–75μm could increase the drug concentration loaded in the tumor, thus exert stronger lethality (77–79). Hepatic lesions in CRLM are less hypervascular in comparison with primary HCC, thus DEB with smaller sizes may have the ability to deliver the drug deeper into the tumor tissue (80).

In addition, there is no standard system for evaluating the efficacy of DEB-TACE. Using different radiological evaluation criteria, such as the Response Evaluation Criteria in Solid Tumors (RECIST) criteria and the Choi criteria, might yield different prognostic results (81). This is because the RECIST criteria only take the long diameter of the tumor but not the density into account. After the TACE procedure, the change in tumor density is greater than the change in the long diameter because the supplying arteries are damaged. Although the RECIST criteria is currently the most commonly used objective method for assessing tumor response, it apparently has shortages compared to the Choi criteria.

Due to the above lack of consensus in both HAIC and TACE, large-scale clinical trials are urgent to provide basis for these treatment approaches of CRLM patients. Herein, we summarized completed and ongoing clinical trials regarding HAIC and TACE for CRLM in **Table 1**. These clinical trials may answer crucial issues and further assist clinical efforts.

**Table 1:** Completed and ongoing clinical trials regarding locoregional interventional chemotherapy for colorectal liver metastasis.

Trial identifier	Country/region	Locoregional therapy	Combined strategy	Estimated/actual enrolled testing patients (n)	Phase	Primary endpoints	Status
NCT05406206	China	Oxaliplatin-5-Fu-based HAIC	Fruquintinib	27	2	PFS	Recruiting
NCT06199232	China	Oxaliplatin-5-Fu-based HAIC/Oxaliplatin-irinotecan-5-Fu-based HAIC	Fruquintinib/Cetuximab& Tislelizumab	47	/	PFS	Recruiting
NCT01891552	Italy	Irinotecan-loaded DEB-TACE	Cetuximab/NA	40	/	PFS	Completed
NCT05794971	China	Irinotecan-loaded DEB-TACE	Regorafenib	63	3	OS	Recruiting
NCT03264716	Taiwan	Doxorubicin-loaded DEB-TACE	5-Fu-based systemic chemotherapy	5	/	PFS	Completed
NCT05511051	China	FOLFOX-based HAIC	Fruquintinib	51	2	OS	Recruiting
NCT02885753	France	Oxaliplatin-based/HAIC	FOLFOX/mFOLFIRINOX & targeted therapy	174	3	PFS	Recruiting
NCT06441565	China	FOLFOX-based HAIC	Fruquintinib	42	2,3	PFS	Recruiting
NCT05103020	Korea	Oxaliplatin-based HAIC	FOLFIRI & targeted therapy	50	2	CRR	Recruiting
NCT03164655	France	Oxaliplatin-based HAIC	FOLFIRI & targeted therapy	10	2	CRR	Completed
NCT04898504	Norway	Floxuridine-based HAIC	/	15	2	OS	Recruiting
NCT04866290	France, Greece	Irinotecan-loaded DEB-TACE	/	105	/	OS	Completed
NCT01348412	France	Raltitrexed-oxaliplatin-based HAIC	Raltitrexed-oxaliplatin systemic chemotherapy	15	2	PFS	Completed

CRR, curative-intent resection rate; DEB, drug-eluting bead; FOLFIRI, irinotecan +leucovorin+fluorouracil; FOLFOX, oxaliplatin+leucovorin+fluorouracil; HAIC, hepatic arterial infusion chemotherapy; mFOLFIRINOX, modified of oxaliplatin+irinotecan+leucovorin+fluorouracil; NA, not applicable; OS, overall survival; PFS, progression-free survival; TACE, transcatheter arterial chemoembolization.

## 4 Transarterial chemotherapy for non-metastatic colorectal cancer

To date, there are only a small amount of related data on locoregional interventional chemotherapy (infusion chemotherapy or chemoembolization) for primary non-metastatic CRC lesions (12, 60, 82–93). Due to the lack of large-scale clinical researches, consensus, and guidelines, this method has been implemented in only a few countries and hospitals (94). Currently, locoregional interventional chemotherapy is mainly applied for locally advanced CRC patients whose basic condition is poor and cannot tolerate the side effects of systemic intravenous chemotherapy. In addition, it can also be used for neoadjuvant therapy, inoperable situations, or postoperative local recurrence. In more than 30% advanced CRC patients, intestinal malignant obstruction is a common complication with high fatality (95). When malignant obstruction occurs, palliative surgery or metal stents imbedding are the most widely used methods. However, they cannot be suitable for patients with poor condition, as well as prevent tumor recurrence. Transarterial chemotherapy combined embolization has been applied in those patients as a safe, effective and feasible approach to prolong the non-obstruction time as well as survival time (93).

Adjuvant chemotherapy can prevent postoperative DM. In those CRC cases treated with surgery and adjuvant chemotherapy but suffered from DM, the resistance of systemic chemotherapy is the main cause. As the DM develops, chemoresistance may have already initiated. Given the high concentration and toxicity of chemotherapeutic drugs, locoregional interventional chemotherapy has the potential to partially reverse chemoresistance. Locally advanced CRC carries a higher risk of DM, so current studies on locoregional interventional chemotherapy in non-metastatic CRC predominantly focus on cases classified as locally advanced CRC.

The current published literature related to locoregional interventional chemotherapy for non-metastatic CRC is summarized in **Table 2**. To our knowledge, the largest-scale study to date is a finished multi-center randomized controlled trial (RCT) by Zhu et al. (NCT00643877) which was led by Zhongshan Hospital of Fudan University, Shanghai, China (90). It concluded that in CRC with stage II-III, the combination of hepatic and regional arterial chemotherapy using FUDR and oxaliplatin greatly decreased the 5-year liver metastasis rate (7% vs. 16%, P<0.001), thus increased the disease-free survival (DFS; 77% vs. 65%, P=0.001) and OS (84% vs. 76%, P=0.005). But limitations exist in this study such as the lack of neoadjuvant therapy in the control group, which might lower the credibility of its results.

**Table 2:** Published literature regarding locoregional interventional chemotherapy for non-metastatic colorectal cancer.

Author/reference	Year	Country/region	Study type	Trial identifier	Tumor site	Patients (n)	Locoregional therapy	Combined strategy	Endpoints
Yang et al. ^a^ (92)	2024	China	Prospective	NCT03601156	Rectum	111	Oxaliplatin-based TACE	Chemoradiotherapy & surgery & adjuvant chemotherapy	pCR, ORR, MPR, DFS, OS
Huang et al. (97)	2023	China	Study protocol	ChiCTR23000 70620	Rectum	/	Irinotecan-raltitrexed-oxaliplatin-based rectal arterial infusion chemotherapy	Chemoradiotherapy + consolidation chemotherapy (TNT) & adjuvant chemotherapy	pCR, cCR, LR, DM
Fan et al. (96)	2023	China	Study protocol	NCT05420584	Rectum	/	Oxaliplatin-raltitrexed-based TACE	Immunotherapy & chemotherapy & surgery	TRG, OS, DFS, pCR
Meng et al.^a^ (12)	2021	China	Prospective	NCT03601156	Rectum	60	Oxaliplatin-based TACE	Chemoradiotherapy & surgery & adjuvant chemotherapy	LR, DM
Meng et al. (60)	2021	China	Case report	/	Rectum	1	Oxaliplatin-based TACE	Chemotherapy	Not mentioned
Zhu et al.^b^ (90)	2021	China	RCT	NCT00643877	Colon, rectum	341	FUDR-oxaliplatin-based colorectal arterial infusion chemotherapy	HAIC & surgery & adjuvant chemotherapy	DFS, LM, OS
Bi et al. (89)	2021	China	Retrospective	/	Colon, rectum	12	DEB-TACE	/	DCR, DFS, OS, PFS
Yang et al.^a^ (88)	2020	China	Prospective	NCT03601156	Rectum	50	Oxaliplatin-based TACE	Chemoradiotherapy & surgery & adjuvant chemotherapy	pCR, ORR
Huang et al. (87)	2019	China	Case report	/	Rectum	9	FOLFOX-based TACE	Chemoradiotherapy & surgery & adjuvant chemotherapy	DFS, OS
Bini et al.^c^ (86)	2016	Italy	Prospective	/	Rectum	12	DEB-TACE	/	RR, OS
Bini et al.^c^ (85)	2015	Italy	Case report	/	Rectum	1	DEB-TACE	Chemotherapy & surgery & adjuvant chemotherapy	Not mentioned
Xu et al.^b^ (84)	2007	China	RCT	/	Colon, rectum	110	FUDR-oxaliplatin-based colorectal arterial infusion chemotherapy	HAIC & surgery	DFS, OS, LM
Kimura et al. (83)	2003	Japan	Case report	/	Rectum	1	Cisplatin-mitomycin-based rectal arterial infusion chemotherapy	Chemoradiotherapy & surgery	Not mentioned
Braun et al. (82)	1997	Ukraine	Retrospective	/	Rectum	52	Adriablastin-fluorouracil-based rectal arterial infusion chemotherapy	Surgery	OS, DM, RFS

^a, b, c^From the same center.

cCR, clinical complete response; DCR, disease control rate; DEB, drug-eluting bead; DFS, disease-free survival; DM, distant metastasis; FOLFOX, oxaliplatin+leucovorin+fluorouracil; FUDR, fluorodeoxyuridine; HAIC, hepatic arterial infusion chemotherapy; LM, liver metastasis; LR, local recurrence; MPR, major pathological response rate; ORR, objective response rate; OS, overall survival; pCR, pathologic complete remission; PFS, progression-free survival; RCT, randomized controlled trial; RFS, relapse-free survival; RR, response rate; TACE, transcatheter arterial chemoembolization; TRG, tumor regression grade; TNT, total neoadjuvant therapy.

According to the studies listed in the table, there is a trend that many new treatment methods are combined with locoregional chemotherapy to treat CRC. Recently, two phase 2 study protocols (NCT05420584 and NCT05957016) have been released regarding the combination of regional chemotherapy and multiple treatments, including systemic chemotherapy, radiotherapy, and immunotherapy for locally advanced rectal cancer (LARC) (96). Another study protocol (ChiCTR2300070620) designed by Huang et al. reported that total neoadjuvant therapy will also be applied concurrently with intra-arterial chemotherapy to LARC patients (97). However, all of these protocols are of single-arm studies, which may be a slight weakness in the study design. Still, the results will be full of expectation because preliminary results (NCT03601156) have proved the efficacy of regional chemotherapy in LARC, and its consecutive clinical trial is ongoing (NCT05957016) (88, 92).

## 5 Combine immunotherapy and locoregional transarterial chemotherapy in colorectal cancer

The potential mechanisms of combining locoregional transarterial chemotherapy with immunotherapy (such as immune checkpoint inhibitors) include enhancing tumor cell sensitivity to immunotherapy through high local concentrations of chemotherapeutic drugs, thereby improving therapeutic efficacy (98). Specific mechanisms involve chemotherapy-induced tumor antigen release and immunogenic cell death, which help enhance the efficacy of immune checkpoint inhibitors. TACE is shown to impact tumor angiogenesis as well as immune function within the tumor microenvironment (TME) (99). Nevertheless, the multifaceted physiological effects of TACE on the TME are uncertain, which encourages the exploration of potential synergies with different anti-tumor drugs to expand its application and improve patient outcomes. In recent years, several key clinical trials have evaluated the use of locoregional transarterial chemotherapy combined with immune checkpoint inhibitors, such as programmed cell death-1 (PD-1) and programmed cell death-ligand 1 (PD-L1) inhibitors, in liver cancer (98). Studies have shown that combined therapy results in significantly longer survival and greater tumor shrinkage compared to monotherapy. The combined therapy strategy has broad clinical practice prospects, including patient selection criteria, optimal drug combinations, and treatment regimens. In NCT05420584 and NCT05957016 trials, immunotherapy is applied both for LARC in combination with locoregional transarterial chemotherapy. Especially in NCT05957016 trial, Tislelizumab, a PD-1 inhibitor, is firstly reported in use for locoregional infusion.

However, some limitations in current researches and trials, such as better control of treatment side effects and optimization of individualized treatment plans, need further exploration. Several successful case studies demonstrated the specific clinical effects and patient outcomes of combining locoregional transarterial chemotherapy with immunotherapy, further proving the feasibility and efficacy of this combined therapy strategy in clinical practice.

## 6 Adverse effects of locoregional chemotherapies for colorectal cancer

Most studies have concluded that HAIC in treating CRLM is a safe method with side effects similar to those of conventional therapies. Symptoms from the digestive system are the most common complications after HAIC treatment with an incidence of up to one-third, including stomatitis, hyperbilirubinemia, elevation of liver enzymes, biliary sclerosis, nausea, vomiting, and diarrhea, etc. (55, 100, 101). Some studies reported that abdominal pain was the only specific side effect of HAIC, which might be due to the drug toxicity to blood vessels, especially when using oxaliplatin (102–104). But some complications related to catheterization, such as hepatic artery occlusion, thrombosis, bile duct necrosis and catheter-related infections, also need to be very highly alerted (47, 105). One example is that due to the long infusion time of approximately 44 hours, 5-Fu-based HAIC might cause a higher incidence of catheter-related thrombosis and infection (106). In summary, although most side effects of HAIC can be addressed by symptomatic treatments, it is necessary to strengthen the monitoring during the course of HAIC.

cTACE is less safe than HAIC in some aspects, although the case fatality rate of cTACE is less than 1% (107). Apart from having the same complications as HAIC, cTACE may result in more post-embolization complications thus prolonging hospital stays (104). Post-embolization syndrome, such as fever, pain, nausea, and vomiting, is a common and controllable reaction to the procedure. In rare cases, the embolization materials used during cTACE may inadvertently affect blood vessels in the surrounding area, leading to complications such as vessel injury or thrombosis (108). In addition, repeated TACE can lead to failure of liver function, thus affecting patient prognosis. The development of DEB-TACE has compensated for the shortcomings of traditional embolizing drugs in terms of action time and safety (109, 110). DEB can facilitate a more uniform distribution of chemotherapy drugs, thereby reducing systemic exposure and minimizing side effects associated with systemic drug administration (108). Therefore, systemic chemoresistance may also be postponed. But in a macro scope, there is no obvious difference in safety between DEB-TACE and cTACE. Several clinical trials and retrospective studies have assessed the safety of DEB-TACE for the treatment of HCC, but there is a lack of studies on the treatment of CRLM.

The application of TACE for primary lesions of CRC has also shown tolerable side effects in published reports. A study by Gao et al. indicated that TACE plus chemotherapy was safer than chemoradiotherapy for LARC patients, especially for reducing the anastomotic leakage (111). Since neoadjuvant radiation may increase the rate of anastomotic leakage after subsequent surgery, especially in rectal cancer patients, studies have confirmed that the single use of chemotherapy in neoadjuvant stage is safer with similar prognosis (112). This finding indicated that TACE may have the potential to replace the role of neoadjuvant radiotherapy in CRC.

Organ perforation is a potential severe adverse event, which is one of the reasons why locoregional chemoembolization of hollow organs is not as common as that of liver cancer. During the growth of tumor tissue, the blood supply of the surrounding tissue is relatively rich, and the central tissue is relatively ischemic. Coupled with the continuous compression and erosion of blood vessels by the tumor, ischemia and necrosis might occur in the center of the tumor. The tumor tissue infiltrates the whole layer of the gastric wall, and the muscle layer is broken, which can sometimes cause perforation. In published studies, no enterobrosis events were reported in patients who received locoregional interventional chemotherapy for primary CRC lesions. A rare syndrome reported in two case studies that delayed perforation occurred in the stomach and duodenum respectively after TACE in HCC patients (113, 114). The cause of such gastrointestinal tract perforation may be arterial ischemia due to embolization, especially after multiple TACEs. This syndrome can occur in such organs with enriched blood supply, so surgeons and nurses should pay more attention to the surgical procedure and post-TACE monitoring in CRC interventional chemotherapy.

In the context of locoregional therapies, the implementation of non-invasive monitoring (NIM) and follow-up is crucial. NIM techniques, such as imaging examinations, multi-model radiomics, and liquid biopsy, can regularly assess the treatment efficacy and promptly observe changes in the tumor (115–117). This is essential for evaluating the effectiveness of the treatment and formulating subsequent treatment plans, especially for those with combined treatments. Besides, NIM can early detect potential complications caused by the treatment, such as vascular injury, tumor bleeding, or local tissue necrosis. Timely intervention measures can be taken to reduce patient risk. Compared to invasive monitoring, NIM processes can improve patient compliance, reduce the psychological burden caused by the treatment, and promote overall health management of the patient. By performing NIM on cancer patients, doctors can adjust treatment plans based on the patient’s actual response, achieving better personalized treatment. This flexibility is important for enhancing treatment effectiveness and reducing side effects. Moreover, NIM plays an important role in patients’ follow-up. Through long-term observation, it is possible to evaluate patients’ safety, survival rates, recurrence rates, and other prognostic indicators, providing a basis for future treatment plans. The conventional NIM methods have intractable limitations, such as the exposure of imaging contrast agents and the limited specificity of serum protein. Advanced molecular detections, like DNA methylation in cell free circulating DNA (cfDNA) and circulating tumor-derived DNA (ctDNA), can be used to track response during metastatic CRC treatment, enabling NIM of tumor burden and drug resistance (115, 118). Furthermore, combining conventional NIM methods and repeated cfDNA or ctDNA assessments will be the directions for future researches to improve prognosis and quality of life in metastatic CRC patients.

## 7 Conclusions

Locoregional transarterial chemotherapy and chemoembolization have shown promising abilities to kill tumor cells, and relieve local symptoms for both primary lesions and liver metastases of CRC, especially when combined with systemic treatments. Meanwhile, the side effects can also be well controlled via various ways. As a palliative treatment, it can become a new addition for advanced patients and prolong survival. As a conversion or neoadjuvant therapy, it can create good conditions for subsequent radical surgery in both liver and primary lesions. On this basis, it is also expected to bring significant improvement to the prognosis. In the future, it is necessary to grasp the specific indications and contraindications, standardize the medication regimen, explore more accurate prognostic predictors, and carry out large-scale prospective studies in multiple centers to further verify the value of locoregional transarterial chemotherapy in CRC.
